# Supplementary material for: Utilization of biochar derived from industrial hemp stalks with various cooling methods for asphalt binder modification
Source: PLoS One. 2025 Jul 2;20(7):e0325943. doi: 10.1371/journal.pone.0325943 (PMC12220996; doi:10.1371/journal.pone.0325943)
Supplement: S1 File — (DOCX) [file pone.0325943.s001.docx]

**THE VALUES USED TO BUILD GRAPHS**

**For Fig 5. Penetration values.**

|  | **Measurement** | | |  |
| --- | --- | --- | --- | --- |
| **Sample** | **1** | **2** | **3** | **Mean** |
| NB | 51.7 | 50.8 | 51.1 | 51.2 |
| 300R5 | 44 | 43.7 | 43.9 | 43.9 |
| 300R10 | 36.0 | 36.8 | 36.5 | 36.4 |
| 300R15 | 32.4 | 30.9 | 31.5 | 31.6 |
| 450R5 | 40.9 | 42.1 | 41.2 | 41.4 |
| 450R10 | 34.2 | 35.1 | 34.5 | 34.6 |
| 450R15 | 30.4 | 30.4 | 31 | 30.6 |
| 600R5 | 40.7 | 40.9 | 41 | 40.9 |
| 600R10 | 37.6 | 36.4 | 36.9 | 37.0 |
| 600R15 | 31.3 | 32.2 | 31.4 | 31.6 |
| 300S5 | 41.4 | 41.8 | 42 | 41.7 |
| 300S10 | 35.6 | 36.5 | 36.4 | 36.2 |
| 300S15 | 32.9 | 33 | 33.2 | 33.0 |
| 450S5 | 40.9 | 42.4 | 41.5 | 41.6 |
| 450S10 | 39.1 | 40 | 38.7 | 39.3 |
| 450S15 | 33 | 34.2 | 34.2 | 33.8 |
| 600S5 | 46.7 | 46.3 | 46.9 | 46.6 |
| 600S10 | 40.4 | 38.9 | 38.4 | 39.2 |
| 600S15 | 35 | 34.6 | 34.3 | 34.6 |

**For Fig 6.** **Softening point values.**

|  | **Measurement** | |  |
| --- | --- | --- | --- |
| **Sample** | **1** | **2** | **Mean** |
| NB | 48 | 48 | 48.0 |
| 300R5 | 50.6 | 50.8 | 50.7 |
| 300R10 | 51.3 | 52.4 | 51.9 |
| 300R15 | 55.5 | 55.8 | 55.7 |
| 450R5 | 50.2 | 51 | 50.6 |
| 450R10 | 52.1 | 52.3 | 52.2 |
| 450R15 | 56 | 57 | 56.5 |
| 600R5 | 49.2 | 49.9 | 49.6 |
| 600R10 | 50.3 | 51.1 | 50.7 |
| 600R15 | 52.8 | 53.6 | 53.2 |
| 300S5 | 51.0 | 51 | 51.0 |
| 300S10 | 51.8 | 51.8 | 51.8 |
| 300S15 | 53.3 | 54 | 53.7 |
| 450S5 | 50.0 | 50.8 | 50.4 |
| 450S10 | 50.8 | 51.2 | 51.0 |
| 450S15 | 53.9 | 54.2 | 54.1 |
| 600S5 | 49.4 | 49.6 | 49.5 |
| 600S10 | 50.8 | 50.8 | 50.8 |
| 600S15 | 53.7 | 53.8 | 53.8 |

**For Fig 7. PI values of asphalt binders.**

| **Sample** | **PI** |
| --- | --- |
| NB | -1.66 |
| 300R5 | -1.32 |
| 300R10 | -1.43 |
| 300R15 | -0.89 |
| 450R5 | -1.47 |
| 450R10 | -1.45 |
| 450R15 | -0.77 |
| 600R5 | -1.75 |
| 600R10 | -1.67 |
| 600R15 | -1.40 |
| 300S5 | -1.35 |
| 300S10 | -1.46 |
| 300S15 | -1.22 |
| 450S5 | -1.50 |
| 450S10 | -1.48 |
| 450S15 | -1.09 |
| 600S5 | -1.48 |
| 600S10 | -1.53 |
| 600S15 | -1.11 |

**Fig 8.** **The relationship between G*/sinδ values determined experimentally and G*/sinδ values predicted by the model**

| **PTEMP** | **COOLING** | **CONT** | **TESTTEMP** | **G*/sinδ (Experimentally)** | **G*/sinδ (Predicted by model)** |
| --- | --- | --- | --- | --- | --- |
| 300 | 1 | 0 | 58 | 3893 | 3963.6 |
| 300 | 1 | 5 | 58 | 5022 | 5161.6 |
| 300 | 1 | 10 | 58 | 6428 | 6621.9 |
| 300 | 1 | 15 | 58 | 9364 | 9334.2 |
| 450 | 1 | 0 | 58 | 3893 | 3963.6 |
| 450 | 1 | 5 | 58 | 5161 | 5279.4 |
| 450 | 1 | 10 | 58 | 7058 | 6663.2 |
| 450 | 1 | 15 | 58 | 9800 | 9434.3 |
| 600 | 1 | 0 | 58 | 3893 | 3963.6 |
| 600 | 1 | 5 | 58 | 5705 | 5385.4 |
| 600 | 1 | 10 | 58 | 6736 | 6683.0 |
| 600 | 1 | 15 | 58 | 8194 | 8693.2 |
| 300 | 2 | 0 | 58 | 3893 | 3822.4 |
| 300 | 2 | 5 | 58 | 4650 | 4688.6 |
| 300 | 2 | 10 | 58 | 6076 | 6053.5 |
| 300 | 2 | 15 | 58 | 8988 | 8824.4 |
| 450 | 2 | 0 | 58 | 3893 | 3822.4 |
| 450 | 2 | 5 | 58 | 4910 | 4806.5 |
| 450 | 2 | 10 | 58 | 5892 | 6094.8 |
| 450 | 2 | 15 | 58 | 8958 | 8924.6 |
| 600 | 2 | 0 | 58 | 3893 | 3822.4 |
| 600 | 2 | 5 | 58 | 4786 | 4912.5 |
| 600 | 2 | 10 | 58 | 6041 | 6114.6 |
| 600 | 2 | 15 | 58 | 8090 | 8183.4 |
| 300 | 1 | 0 | 64 | 1765 | 1762.6 |
| 300 | 1 | 5 | 64 | 2322 | 2376.8 |
| 300 | 1 | 10 | 64 | 3039 | 3091.5 |
| 300 | 1 | 15 | 64 | 4509 | 4447.3 |
| 450 | 1 | 0 | 64 | 1765 | 1762.6 |
| 450 | 1 | 5 | 64 | 2465 | 2494.6 |
| 450 | 1 | 10 | 64 | 3124 | 3132.9 |
| 450 | 1 | 15 | 64 | 4681 | 4547.5 |
| 600 | 1 | 0 | 64 | 1765 | 1762.6 |
| 600 | 1 | 5 | 64 | 2646 | 2600.6 |
| 600 | 1 | 10 | 64 | 3142 | 3152.7 |
| 600 | 1 | 15 | 64 | 3715 | 3806.3 |
| 300 | 2 | 0 | 64 | 1765 | 1767.4 |
| 300 | 2 | 5 | 64 | 2154 | 2049.8 |
| 300 | 2 | 10 | 64 | 2785 | 2669.1 |
| 300 | 2 | 15 | 64 | 3948 | 4083.6 |
| 450 | 2 | 0 | 64 | 1765 | 1767.4 |
| 450 | 2 | 5 | 64 | 2221 | 2167.6 |
| 450 | 2 | 10 | 64 | 2625 | 2710.5 |
| 450 | 2 | 15 | 64 | 4038 | 4183.7 |
| 600 | 2 | 0 | 64 | 1765 | 1767.4 |
| 600 | 2 | 5 | 64 | 2155 | 2273.6 |
| 600 | 2 | 10 | 64 | 2772 | 2730.3 |
| 600 | 2 | 15 | 64 | 3620 | 3442.6 |
| 300 | 1 | 0 | 70 | 858 | 789.8 |
| 300 | 1 | 5 | 70 | 1126 | 1110.3 |
| 300 | 1 | 10 | 70 | 1486 | 1512.4 |
| 300 | 1 | 15 | 70 | 2246 | 2230.4 |
| 450 | 1 | 0 | 70 | 858 | 789.8 |
| 450 | 1 | 5 | 70 | 1218 | 1228.1 |
| 450 | 1 | 10 | 70 | 1478 | 1553.8 |
| 450 | 1 | 15 | 70 | 2138 | 2330.5 |
| 600 | 1 | 0 | 70 | 858 | 789.8 |
| 600 | 1 | 5 | 70 | 1306 | 1334.1 |
| 600 | 1 | 10 | 70 | 1494 | 1573.6 |
| 600 | 1 | 15 | 70 | 1766 | 1589.4 |
| 300 | 2 | 0 | 70 | 858 | 926.2 |
| 300 | 2 | 5 | 70 | 1028 | 914.9 |
| 300 | 2 | 10 | 70 | 1356 | 1221.6 |
| 300 | 2 | 15 | 70 | 1863 | 1998.2 |
| 450 | 2 | 0 | 70 | 858 | 926.2 |
| 450 | 2 | 5 | 70 | 1034 | 1032.8 |
| 450 | 2 | 10 | 70 | 1241 | 1262.9 |
| 450 | 2 | 15 | 70 | 1904 | 2098.4 |
| 600 | 2 | 0 | 70 | 858 | 926.2 |
| 600 | 2 | 5 | 70 | 1047 | 1138.8 |
| 600 | 2 | 10 | 70 | 1352 | 1282.8 |
| 600 | 2 | 15 | 70 | 1687 | 1357.2 |

**For Fig 9. (a) CAI. (b) PAI and (c) RAI aging indices.**

| **CAI** | | | |  | **PAI** | | | |  | **RAI** | | | |
| --- | --- | --- | --- | --- | --- | --- | --- | --- | --- | --- | --- | --- | --- |
| **Sample** | **58°C** | **64°C** | **70°C** |  | **Sample** | **58°C** | **64°C** | **70°C** |  | **Sample** | **58°C** | **64°C** | **70°C** |
| NB | 3.38 | 3.08 | 3.09 |  | NB | 0.93 | 0.94 | 0.95 |  | NB | 3.44 | 3.11 | 3.11 |
| 300R5 | 2.14 | 2.12 | 1.99 |  | 300R5 | 0.95 | 0.96 | 0.97 |  | 300R5 | 2.16 | 2.13 | 2.00 |
| 300R10 | 2.46 | 2.39 | 2.13 |  | 300R10 | 0.95 | 0.96 | 0.97 |  | 300R10 | 2.48 | 2.4 | 2.13 |
| 300R15 | 2.42 | 2.14 | 2.04 |  | 300R15 | 0.95 | 0.96 | 0.97 |  | 300R15 | 2.45 | 2.16 | 2.05 |
| 300S5 | 2.38 | 2.26 | 2.29 |  | 300S5 | 0.95 | 0.96 | 0.97 |  | 300S5 | 2.40 | 2.27 | 2.3 |
| 300S10 | 2.21 | 2.1 | 2.07 |  | 300S10 | 0.95 | 0.97 | 0.98 |  | 300S10 | 2.23 | 2.11 | 2.07 |
| 300S15 | 2.23 | 2.57 | 2.2 |  | 300S15 | 0.95 | 0.96 | 0.97 |  | 300S15 | 2.26 | 2.59 | 2.21 |

**For Fig 10.** **(a) Jnr and R values at 0.1 kPa**

| **Sample** | **Jnr_0.1_** | **R_0.1_** |
| --- | --- | --- |
| NB | 1.4 | 10 |
| 300R5 | 1.63 | 9 |
| 300R10 | 1.11 | 15 |
| 300R15 | 0.72 | 19 |
| 300S5 | 1.85 | 9 |
| 300S10 | 1.64 | 13 |
| 300S15 | 0.91 | 15 |

**For Fig 10.** **(b) Jnr and R values at 3.2 kPa. and traffic class**

| **Sample** | **Jnr_3.2_** | **R_3.2_** |
| --- | --- | --- |
| NB | 1.64 | 3 |
| 300R5 | 1.86 | 3 |
| 300R10 | 1.37 | 4 |
| 300R15 | 0.92 | 6 |
| 300S5 | 2.15 | 2 |
| 300S10 | 2.01 | 3 |
| 300S15 | 1.05 | 3 |

**For Fig 11. Jnr_diff_ values.**

| **Sample** | **Jnr_diff_** |
| --- | --- |
| NB | 17 |
| 300R5 | 14 |
| 300R10 | 23 |
| 300R15 | 28 |
| 300S5 | 16 |
| 300S10 | 22 |
| 300S15 | 16 |
