# Supplementary material for: Utilization of biochar derived from industrial hemp stalks with various cooling methods for asphalt binder modification
Source: PLoS One. 2025 Jul 2;20(7):e0325943. doi: 10.1371/journal.pone.0325943 (PMC12220996; doi:10.1371/journal.pone.0325943)
Supplement: Attachment — Submitted filename: Response to Reviewers.docx [file pone.0325943.s003.docx]

**Explanation of the corrections that have been made by following the reviewers comments.**

*Many thanks for useful comments to our paper, and good advice for changes!*

**Reviewer #1**

1. Introduction: This section should include previous studies on variation of pyrolysis conditions, and should also explain the significance of the pyrolysis parameters (temperature and rate of cooling). Authors are encouraged to check for papers from chemical / energy engineering domain to enhance the quality of the introduction.

To explain the importance of pyrolysis parameters, the section of the introduction dealing with pyrolysis is re-written as follows:

*“…source or additive material.* *Pyrolysis, the most commonly used and extensively studied process for biochar production, involves the thermal decomposition of biomass at elevated temperatures in an oxygen-free environment [6–8]. During pyrolysis, the biomass undergoes thermal decomposition, releasing volatile compounds and leaving behind a solid residue known as biochar. Biochar can be produced from a wide range of biomass, including agricultural waste, forestry waste, and municipal and industrial organic waste. The yield and properties of the biochar depend on various factors such as the type of biomass, the heating rate, pyrolysis temperature, and the residence time [9]. There are three different pyrolysis processes: slow, fast and flash pyrolysis. Slow pyrolysis yields more biochar products at low temperature and low heating rate, while fast pyrolysis yields higher bio-oil product at high temperature such and high heating rate. In flash pyrolysis, the heating rate is higher than in fast pyrolysis and the processing time is quite short [10]. In slow pyrolysis, characterized by low reaction temperatures and heating rates, the biochar yield is maximized, resulting in the production of high-quality biochar [11, 12]. Most of the previous laboratory scale research on slow pyrolysis investigated the effect of type of biomass, pyrolysis temperature, heating rate, and residence time, as the main operating variables that affected the biochar yield and properties [8, 13-19].* *Biochar derived from different…”*

The following references have been added to manuscript.

6. Tan H, Lee CT, Ong PY, Wong KY, Bong CPC, Li C, et al. A Review On The Comparison Between Slow Pyrolysis And Fast Pyrolysis On The Quality Of Lignocellulosic And Lignin-Based Biochar. IOP Conf Ser Mater Sci Eng. 2021;1051: 012075. doi:10.1088/1757-899X/1051/1/012075

7. Ng HS, Kee PE, Yim HS, Chen PT, Wei YH, Chi-Wei Lan J. Recent advances on the sustainable approaches for conversion and reutilization of food wastes to valuable bioproducts. Bioresour Technol. 2020;302: 122889. doi:10.1016/j.biortech.2020.122889

9. Zhou L. A Review of Biomass-Derived Biochar and Its Potential in Asphalt Pavement Engineering. Materials Science- Poland. 2024;42: 81–99. doi:10.2478/msp-2024-0022

10. Lee SY, Sankaran R, Chew KW, Tan CH, Krishnamoorthy R, Chu D-T, et al. Waste to bioenergy: a review on the recent conversion technologies. Bmc Energy. 2019;1. doi:10.1186/s42500-019-0004-7

11. Onay O, Kockar OM. Slow, fast and flash pyrolysis of rapeseed. Renew Energy. 2003;28: 2417–2433. doi:10.1016/S0960-1481(03)00137-X

12. Maqsood T, Dai J, Zhang Y, Guang M, Li B. Pyrolysis of plastic species: A review of resources and products. J Anal Appl Pyrolysis. 2021;159. doi:10.1016/j.jaap.2021.105295

13. Premchand P, Demichelis F, Chiaramonti D, Bensaid S, Fino D. Biochar production from slow pyrolysis of biomass under CO2 atmosphere: A review on the effect of CO2 medium on biochar production, characterisation, and environmental applications. J Environ Chem Eng. 2023;11: 110009. doi:10.1016/j.jece.2023.110009

14. Leng L, Huang H. An overview of the effect of pyrolysis process parameters on biochar stability. Bioresour Technol. 2018;270: 627–642. doi:10.1016/j.biortech.2018.09.030

15. Tag AT, Duman G, Ucar S, Yanik J. Effects of feedstock type and pyrolysis temperature on potential applications of biochar. J Anal Appl Pyrolysis. 2016;120: 200–206. doi:10.1016/j.jaap.2016.05.006

16. Elnour AY, Alghyamah AA, Shaikh HM, Poulose AM, Al-Zahrani SM, Anis A, et al. Effect of Pyrolysis Temperature on Biochar Microstructural Evolution, Physicochemical Characteristics, and Its Influence on Biochar/Polypropylene Composites. Applied Sciences 2019, Vol 9, Page 1149. 2019;9: 1149. doi:10.3390/APP9061149

17. Tomczyk A, Sokołowska Z, Science PB-R in E, 2020 undefined. Biochar physicochemical properties: pyrolysis temperature and feedstock kind effects. Springer. 2020;19: 191–215. doi:10.1007/s11157-020-09523-3

18. Li C, Hayashi J ichiro, Sun Y, Zhang L, Zhang S, Wang S, et al. Impact of heating rates on the evolution of function groups of the biochar from lignin pyrolysis. J Anal Appl Pyrolysis. 2021;155. doi:10.1016/j.jaap.2021.105031

19. Zhao B, O’Connor D, Zhang J, Peng T, Shen Z, Tsang DCW, et al. Effect of pyrolysis temperature, heating rate, and residence time on rapeseed stem derived biochar. J Clean Prod. 2018;174: 977–987. doi:10.1016/j.jclepro.2017.11.013

2. In Introduction section, briefly add a paragraph to explain the need of the study and the novelty.

The last part of the Introduction has been revised as follows to emphasize the study's significance and originality:

*Factors affecting the yield and properties of biochar have also been taken into account in previous studies on biochar-modified asphalt binders and their mixtures [8]. No detailed study has been conducted on the cooling of biochar obtained after pyrolysis. In some studies, biochar was extracted from the steel reactor only after it had cooled to room temperature following the pyrolysis process, a procedure called as slow cooling, whereas other studies did not specify the cooling method. In this study, after pyrolysis, the hot biochar was immediately removed from the steel reactor, rapidly cooled by immersing it in water at 10°C, and then dried. This process was termed rapid cooling. In the study, biochar obtained by both methods was used in the experiments. The aim of the study was to determine the optimum pyrolysis temperature and cooling conditions for producing biochar from industrial hemp stalks to enhance asphalt binder performance. Additionally, the study evaluated the potential of industrial hemp stalks as a viable alternative in the pavement sector by analyzing the temperature sensitivity and high- and low-temperature performance of the binder with biochar incorporation. Although there are studies on biochar production from industrial hemp stalk for various purposes [39-42], as far as the authors are aware, no detailed research has been conducted on the performance properties of the asphalt binder incorporating industrial hemp stalk biochar.*

The following references have been added to manuscript.

39. Du J, Zhang F, Hu J, Yang S. Co-pyrolysis of industrial hemp stems and waste plastics into biochar-based briquette: Product characteristics and reaction mechanisms. Fuel Processing Technology. 2023;247: 107793. doi:10.1016/j.fuproc.2023.107793

40. Voglar J, Prašnikar A, Moser K, Carlon E, Schwabl M, Likozar B. Pyrolysis of industrial hemp biomass from contaminated soil phytoremediation: Kinetics, modelling transport phenomena and biochar-based metal reduction. Thermochim Acta. 2024;742: 179899. doi:https://doi.org/10.1016/j.tca.2024.179899

41. Marrot L, Candelier K, Valette · Jérémy, Lanvin · Charline, Horvat B, Legan · Lea, et al. Valorization of hemp stalk waste through thermochemical conversion for energy and electrical applications. Waste Biomass Valorization. 2022;13: 2267–2285. doi:10.1007/s12649-021-01640-6

42. Atoloye ID IA, Adesina IS, Sharma H, Subedi KI, Liang C-L, Shahbazi A, et al. Hemp biochar impacts on selected biological soil health indicators across different soil types and moisture cycles. PLoS One. 2022;17. doi:10.1371/journal.pone.0264620

***Note:*** *The pyrolysis methods include slow pyrolysis, fast pyrolysis, and flash pyrolysis. The rapid cooling process mentioned in our study is a distinct operation from the fast pyrolysis method and is performed after biochar production. Therefore, the terms "fast pyrolysis" and "rapid cooling" refer to different concepts and should not be conflated.*

3. L74-77: Please do not include the conclusions of your study in the introduction section.

The conclusions of our study given in line 74-77 (in old version) have been removed from the introduction section. In the new version of the manuscript, the results of our study are not given in the introduction.

*L74-77: The optimal production parameters of biochar were determined as a temperature of 300 °C combined with rapid cooling. The incorporation of biochar enhanced the aging resistance and high-temperature performance of bituminous binders, although it resulted in a slight reduction in low-temperature performance.*

4. Para 2.2: Please add several pics to show the processing of the material used in pyrolysis, pyrolysis reactor (actual picture or a flow chart), process of slow and rapid cooling, and any other relevant picture.

With respect to the reviewer comment, new figure is added in the text to explain graphically the pyrolysis process. The following sentences in red have been added to section 2.3.

*“…The pyrolysis system employed for biochar production illustrated in Fig 1. Allowing the biochar to cool to room temperature within the steel reactor after the pyrolysis process is referred to as slow cooling, while rapidly cooling the biochar by immersing it in cold water immediately after its removal from the steel reactor is referred to as rapid cooling. The processes of the rapid cooling and slow cooling are schematically shown in Fig 2. After…”*

Fig 1. (a) Pyrolysis system and (b) schematic illustration.

Fig 2. (a) Rapid cooling process and (b) slow cooling process.

5. L187-188: Please add the results of previous studies in Table 3 in support of your statement that “elemental analysis results…were found similar to those of previous studies.”

Sentences in lines 187-188 (now line 215-220) were re-written with additional reference to include the results of previous studies and compared with the results found in our study,and the rewritten part is marked in red.

“*Table 3 presents the elemental analysis results of biochars produced from industrial hemp stalks of varying sizes under different pyrolysis conditions. The elemental analysis results indicated that raw hemp stalk with a particle size smaller than 0.25 mm contained 42.16% carbon, 5.995% hydrogen, and 0.257% nitrogen, while raw hemp stalk with a particle size of 10-30 mm contained 45.63% carbon, 6.435% hydrogen, and 0.36% nitrogen. Our findings consistent with the data reported in the literature, emphasizing that the carbon content of raw hemp biomass ranges from 45% to 49%, hydrogen from 5.6% to 6.2%, and nitrogen from 0.3% to 1.3% [41, 59, 60].*

The following references have been added to manuscript.

*41. Marrot L, Candelier K, Valette · Jérémy, Lanvin · Charline, Horvat B, Legan · Lea, et al. Valorization of hemp stalk waste through thermochemical conversion for energy and electrical applications. Waste Biomass Valorization. 2022;13: 2267–2285. doi:10.1007/s12649-021-01640-6.*

*59. Prade T, Finell M, Svensson SE, Mattsson JE. Effect of harvest date on combustion related fuel properties of industrial hemp (Cannabis sativa L.). Fuel. 2012;102: 592–604. doi:10.1016/j.fuel.2012.05.045*

6. Para 4.1: In Para 3.1, you mention conducting CHNS analysis; however the results presented are for CHN.

*As a result of the elemental analysis, S was found to be zero, so it is not given in the table. The results found are consistent with previous results.* *Since S was found to be zero as a result of the elemental analysis, the term “CHN analysis” is used instead of “CHNS analysis” throughout the article.*

*Pannipa Chaowana, Warinya Hnoocham, Sumate Chaiprapat, Piyawan Yimlamai, Korawit Chitbanyong, Kapphapaphim Wanitpinyo, Tanapon Chaisan, Yupadee Paopun, Sawitree Pisutpiched, Somwang Khantayanuwong, Buapan Puangsin, Utilization of hemp stalk as a potential resource for bioenergy, Materials Science for Energy Technologies,Volume 7, 2024, Pages 19-28, https://doi.org/10.1016/j.mset.2023.07.001.*

7. Para 4.1: Please add some scientific insights into the variation of the results. What causes changes in CHN contents on slow / rapid cooling? What happens when the pyrolysis temperature is increased?

To explain the variation of the results clearly, in Section “4.1. Elemental and SEM Analysis Results” the following with red colour was added:

*“Table 3 shows that the increase in the carbon amount of biochar obtained from the pyrolysis of small-sized biomass is high. The carbon content of hemp stalk biochar with a particle size smaller than 0.25 mm was found to range from 69.73% to 95.93%, while that of biochar with a particle size of 10–30 mm ranged from 65.5% to 83.85%, depending on the pyrolysis temperature and the cooling conditions. Changing the pyrolysis temperature and cooling conditions did not lead to a meaningful change in the observed increase in carbon. Hydrogen content exhibits an inverse relationship with temperature, decreasing as pyrolysis progresses. For small particles, hydrogen content drops from 3.884% at 300°C to 1.62% at 600°C under rapid cooling conditions. This reduction is attributed to the volatilization of hydrogen-containing compounds and the progressive formation of aromatic structures, which lead to lower H/C ratios and increased thermal stability of the biochar. Similarly, nitrogen content decreases with increasing temperature as nitrogenous compounds volatilize into gaseous emissions such as NH₃ and NOx.”*

8. Table 4: The table is data intensive; suggest presenting them in the form of a graph.

As seen in the figure below, since the number of data is large and it is difficult to read the graph, it was thought that it would be appropriate to give the data in a table and the data is given in the Table *4.*

9. Para 4.4.1: Why did you use ‘dummy’ variables for numerical data (pyrolysis temperature, biochar content, DSR temperature)? Dummy variable is justified for cooling condition as the levels are not numeric.

We would like to thank to the reviewer for pointing out this mistake. During the statistical analysis, level sets were used only for the cooling condition, but level sets were also assigned to other independent variables by mistake in Table 6 and the error was corrected now. Table 6 was rearranged as follows. The following change marked with red color has been made in the manuscript:

*Since the cooling condition one of the independent variables addressed is qualitative variable, "level set" was assigned to this variable to observe its contribution to the model.*

**Table 6.** **Variables used in variance analysis.**

| **Independent variables** | **Level** | **Definition** |
| --- | --- | --- |
| Production Temperature  (PTEMP) | 300 °C  450 °C  600 °C | Biochar production temperature |
| Cooling Condition  (COOLING) | Rapid (1)  Slow (2) | Biochar cooling conditions |
| Biochar Utilisation Content (CONT) | 0  %5  %10  %15 | Used biochar contents |
| DSR Test Temperature  (TESTTEM) | 58 °C  64 °C  70 °C | DSR test temperatures |
| **Dependent variables** | **Definition** | |
| G*/sinδ | Rutting parameter | |

10. Revise your statistical model by removing the non-significant terms.

Statistical model was revised by removing the non-significant terms. Table 7 and Equation 2 were rearranged as follows.

**Table 7.** **Statistical model details.**

| **Statistical parameter** | **Values** | **Definition** |
| --- | --- | --- |
| R^2^ | 0.997 | (P_r_>F) <0,0001 (significant) |
| RMSE, kPa | 0.0171 |  |
| **Parameter/Interaction** | **P_r_>F** | **Definition** |
| PTEMP | 0.0122 | S |
| COOLING | <0.0001 | S |
| CONT | <0.0001 | S |
| TESTTEMP | <0.0001 | S |
| PTEMP*CONT | <0.0001 | S |
| COOLING*CONT | 0.0024 | S |
| COOLING*TESTTEMP | 0.0265 | S |
| CONT*TESTTEMP | <0.0001 | S |

G*/sinδ = μ + α1 PTEMP + α2COOLING + α3CONT + α4TESTTEM + α5PTEMP*CONT +

+ α6COOLING*CONT + α7COOLING*TESTTEM + α8CONT*TESTTEM

**Reviewer #2**

1. Line 46: Add a reference

The following references have been added after the sentence "The primary applications of biochars currently lie within the agricultural sector." on line 45.

***References:***

*20. Allohverdi T, Mohanty AK, Roy P, Misra M. A review on current status of biochar uses in agriculture. Molecules. 2021;26. doi:10.3390/molecules26185584*

*21. Deshani Igalavithana A, Mandal S, Khan Niazi N, Vithanage M, Parikh SJ, D Mukome FN, et al. Advances and future directions of biochar characterization methods and applications. Crit Rev Environ Sci Technol. 2017;47: 2275–2330. doi:10.1080/10643389.2017.1421844*

*22. Bandara T, Franks A, Xu J, Bolan N, Wang H, Tang C. Chemical and biological immobilization mechanisms of potentially toxic elements in biochar-amended soils. Crit Rev Environ Sci Technol. 2020;2020: 903–978. doi:10.1080/10643389.2019.1642832*

*23. Carvalho ML, de Moraes MT, Cerri CEP, Cherubin MR. Biochar amendment enhances water retention in a tropical sandy soil. Agriculture (Switzerland). 2020;10. doi:10.3390/agriculture10030062.*

*24. Carvalho ML, de Moraes MT, Cerri CEP, Cherubin MR. Biochar amendment enhances water retention in a tropical sandy soil. Agriculture (Switzerland). 2020;10. doi:10.3390/agriculture10030062.*

The following references have been added after the sentence "In the construction industry, biochars have predominantly been investigated as a partial substitute for cement in mortar or concrete." in Line 46

***References***

*25. Legan M, Gotvajn AŽ, Zupan K. Potential of biochar use in building materials. J Environ Manage. 2022;309. doi:10.1016/j.jenvman.2022.114704*

*26. Maljaee H, Madadi R, Paiva H, Tarelho L, Ferreira VM. Incorporation of biochar in cementitious materials: A roadmap of biochar selection. Constr Build Mater. 2021;283. doi:10.1016/j.conbuildmat.2021.122757.*

*27. Afriyie Mensah R, Shanmugam V, Narayanan S, Razavi N, Ulfberg A, Blanksvärd T, et al. Biochar-Added cementitious materials—A review on mechanical, thermal, and environmental properties. Sustainability. 2021;13. doi:10.3390/su13169336.*

*28. Danish A, Ali Mosaberpanah M, Usama Salim M, Ahmad N, Ahmad F, Ahmad A. Reusing biochar as a filler or cement replacement material in cementitious composites: A review. Constr Build Mater. 2021;300. doi:10.1016/j.conbuildmat.2021.124295.*

*29. Maljaee H, Paiva H, Madadi R, Tarelho LAC, Morais M, Ferreira VM. Effect of cement partial substitution by waste-based biochar in mortars properties. Constr Build Mater. 2021;301. doi:10.1016/j.conbuildmat.2021.124074*

*30. Tan K, Qin Y, Wang J. Evaluation of the properties and carbon sequestration potential of biochar-modified pervious concrete. Constr Build Mater. 2022;314. doi:10.1016/j.conbuildmat.2021.125648*

2. Line 103: Sentence seems to have a grammatical problem

It has been corrected.

3. Line 161: Add a reference for that statement

The following reference was added after the statement "The maximum G*.sinδ value of PAV-aged asphalt binders is limited to 5000 kPa." in Line 161 (Line 188 in new version)

.

***Reference:***

*52. McGennis R, Anderson R, Kennedy T. Background of SUPERPAVE asphalt mixture design and analysis. 1995 [cited 19 Mar 2025]. Available: https://rosap.ntl.bts.gov/view/dot/42577*

4. Under section 4.2, what do you think caused those results

Biochar mainly presents a porous and fibrous structure. Biochar with a porous fibrous structure is considered to cause the results given in section 4.2. Therefore, the following sentence in red has been added to section 4.2.

*“…This indicates that the addition of biochar, especially obtained under rapid cooling condition, can reduce the temperature susceptibility of asphalt binders and improve their high temperature performance. Due to its inherently porous and fibrous structure [63], biochar strengthens the asphalt binder, contributing to improved thermal stability.”*

**Reviewer #3**

1. It is needed to revise the title of the article as it does not reflect the paper’s content well.

The title of the article has been revised to more accurately reflect the content, in accordance with the reviewer's suggestion, and is presented below.

*Utilization of Biochar Derived from Industrial Hemp Stalks with Various Cooling Methods for Asphalt Binder Modification*

2. In the beginning of the abstract, it is mentioned “This study aimed to determine the optimal pyrolysis temperature and cooling conditions to produce biochar from industrial hemp stalks” but the authors did not state the optimal temperature and cooling conditions.

In “Abstract” section we stated the optimal temperature and cooling conditions as following sentence.

*The optimal production conditions for biochar, utilized in the modification of asphalt binders to achieve better rutting resistance, have been identified as a combination of a pyrolysis temperature of 300°C and rapid cooling.”*

3. The abstract must be written quantitatively to reflect the effect of adding biochar to bitumen by using increasing and decreasing values. For example, the authors stated that “It was determined that the biochar additive had a minimal negative effect on the low-temperature performance of the binder”. It is required to state a specific value for “minimal negative effect”.

*Abstract is re-arranged. Due to the number of asphalt binder types and the abundance of data, increasing and decreasing values reflecting the effect of biochar addition to bitumen are given in the manuscript. In this way, there are articles with a summary section and similarly, articles with a summary section are given below.*

*a. Jure Voglar, Anže Prašnikar, Konstantin Moser, Elisa Carlon, Manuel Schwabl, Blaž Likozar, Pyrolysis of industrial hemp biomass from contaminated soil phytoremediation: Kinetics, modelling transport phenomena and biochar-based metal reduction, Thermochimica Acta, Volume 742, 2024, 179899, https://doi.org/10.1016/j.tca.2024.179899.*

*b. Y. Edwards, Y. Tasdemir, U. Isacsson, Rheological effects of commercial waxes and polyphosphoric acid in bitumen 160/220 – high and medium temperature performance, Construction and Building Materials,*

*Volume 21, Issue 10, 2007, Pages 1899-1908, https://doi.org/10.1016/j.conbuildmat.2006.07.012.*

#### c. Influence of Commercial Waxes on Bitumen Aging Properties Ylva Edwards, Yuksel Tasdemir, and Ulf Isacsson Energy & Fuels **2005** 19 (6), 2519-2525 DOI: 10.1021/ef050166r

*d. Atoloye IA, Adesina IS, Sharma H, Subedi K, Liang CL(, et al. (2022) Hemp biochar impacts on selected biological soil health indicators across different soil types and moisture cycles. PLOS ONE 17(2): e0264620.* [*https://doi.org/10.1371/journal.pone.0264620*](https://doi.org/10.1371/journal.pone.0264620)

4. As the main research gap, in the last paragraph of the introduction section, the authors stated “The primary focus of this study, unlike previous ones, is to determine the optimal production temperature and cooling conditions for pyrolysis in biochar production.” While it cannot be an appropriate research gap, it is not aligned with the literature review that the authors presented in previous paragraphs. There are lots of studies that focus on determining optimal pyrolysis temperature and cooling conditions. It is necessary to specify the main research gap that motivated the authors to conduct this study.

The last part of the Introduction has been revised as follows to emphasize the study's significance and originality:

*Factors affecting the yield and properties of biochar have also been taken into account in previous studies on biochar-modified asphalt binders and their mixtures [8]. No detailed study has been conducted on the cooling of biochar obtained after pyrolysis. In some studies, biochar was extracted from the steel reactor only after it had cooled to room temperature following the pyrolysis process, a procedure called as slow cooling, whereas other studies did not specify the cooling method. In this study, after pyrolysis, the hot biochar was immediately removed from the steel reactor, rapidly cooled by immersing it in water at 10°C, and then dried. This process was termed rapid cooling. In the study, biochar obtained by both methods was used in the experiments. The aim of the study was to determine the optimum pyrolysis temperature and cooling conditions for producing biochar from industrial hemp stalks to enhance asphalt binder performance. Additionally, the study evaluated the potential of industrial hemp stalks as a viable alternative in the pavement sector by analyzing the temperature sensitivity and high- and low-temperature performance of the binder with biochar incorporation. Although there are studies on biochar production from industrial hemp stalk for various purposes [39-42], as far as the authors are aware, no detailed research has been conducted on the performance properties of the asphalt binder incorporating industrial hemp stalk biochar.*

The following references have been added to manuscript.

39. Du J, Zhang F, Hu J, Yang S. Co-pyrolysis of industrial hemp stems and waste plastics into biochar-based briquette: Product characteristics and reaction mechanisms. Fuel Processing Technology. 2023;247: 107793. doi:10.1016/j.fuproc.2023.107793

40. Voglar J, Prašnikar A, Moser K, Carlon E, Schwabl M, Likozar B. Pyrolysis of industrial hemp biomass from contaminated soil phytoremediation: Kinetics, modelling transport phenomena and biochar-based metal reduction. Thermochim Acta. 2024;742: 179899. doi:https://doi.org/10.1016/j.tca.2024.179899

41. Marrot L, Candelier K, Valette · Jérémy, Lanvin · Charline, Horvat B, Legan · Lea, et al. Valorization of hemp stalk waste through thermochemical conversion for energy and electrical applications. Waste Biomass Valorization. 2022;13: 2267–2285. doi:10.1007/s12649-021-01640-6

42. Atoloye ID IA, Adesina IS, Sharma H, Subedi KI, Liang C-L, Shahbazi A, et al. Hemp biochar impacts on selected biological soil health indicators across different soil types and moisture cycles. PLoS One. 2022;17. doi:10.1371/journal.pone.0264620

***Note:*** *The pyrolysis methods include slow pyrolysis, fast pyrolysis, and flash pyrolysis. The rapid cooling process mentioned in our study is a distinct operation from the fast pyrolysis method and is performed after biochar production. Therefore, the terms "fast pyrolysis" and "rapid cooling" refer to different concepts and should not be conflated.*

5. It is required that the authors expand their literature review to avoid claiming some statement like this “no prior study in the literature has utilized industrial hemp for the production of biochar.” For example, take a look at the following references:

Du, J., Zhang, F., Hu, J., Yang, S., Liu, H., & Wang, H. (2023). Co-pyrolysis of industrial hemp stems and waste plastics into biochar-based briquette: Product characteristics and reaction mechanisms. Fuel Processing Technology, 247, 107793.

Voglar, J., Prašnikar, A., Moser, K., Carlon, E., Schwabl, M., & Likozar, B. (2024). Pyrolysis of industrial hemp biomass from contaminated soil phytoremediation: kinetics, modelling transport phenomena and biochar-based metal reduction. Thermochimica acta, 742, 179899.

Our sentence was “Notably, no prior study in the literature has utilized industrial hemp for the production of biochar **intended for binder modification**.”. In our sentence, it is intended to express that no detailed research has been conducted on the use of biochar produced from industrial hemp in asphalt binder modification. There are studies on the production of biochar from hemp for different purposes. To make the sentence clearer, it is re-written in Introduction section as:

*Although there are studies on biochar production from industrial hemp stalk for various purposes [39-42], as far as the authors are aware, no detailed research has been conducted on the performance properties of the asphalt binder* ***incorporating industrial hemp stalk biochar****.*

The following references have been added to manuscript.

39. Du J, Zhang F, Hu J, Yang S. Co-pyrolysis of industrial hemp stems and waste plastics into biochar-based briquette: Product characteristics and reaction mechanisms. Fuel Processing Technology. 2023;247: 107793. doi:10.1016/j.fuproc.2023.107793

40. Voglar J, Prašnikar A, Moser K, Carlon E, Schwabl M, Likozar B. Pyrolysis of industrial hemp biomass from contaminated soil phytoremediation: Kinetics, modelling transport phenomena and biochar-based metal reduction. Thermochim Acta. 2024;742: 179899. doi:https://doi.org/10.1016/j.tca.2024.179899

41. Marrot L, Candelier K, Valette · Jérémy, Lanvin · Charline, Horvat B, Legan · Lea, et al. Valorization of hemp stalk waste through thermochemical conversion for energy and electrical applications. Waste Biomass Valorization. 2022;13: 2267–2285. doi:10.1007/s12649-021-01640-6

42. Atoloye ID IA, Adesina IS, Sharma H, Subedi KI, Liang C-L, Shahbazi A, et al. Hemp biochar impacts on selected biological soil health indicators across different soil types and moisture cycles. PLoS One. 2022;17. doi:10.1371/journal.pone.0264620

6. The results should not be mentioned in the introduction section as the authors stated “The optimal production parameters of biochar were determined as a temperature of 300 °C combined with rapid cooling”.

The conclusions of our study given in line 74-77 have been removed from the introduction section.

*L74-77: The optimal production parameters of biochar were determined as a temperature of 300 °C combined with rapid cooling. The incorporation of biochar enhanced the aging resistance and high-temperature performance of bituminous binders, although it resulted in a slight reduction in low-temperature performance*

7. The authors must state the objectives of the study at the of the introduction section.

*The following sentences have been added to the last part of the Introduction section.*

“…*The aim of the study was to determine the optimum pyrolysis temperature and cooling conditions for producing biochar from industrial hemp stalks to enhance asphalt binder performance. Additionally, the study evaluated the potential of industrial hemp stalks as a viable alternative in the pavement sector by analyzing the temperature sensitivity and high- and low-temperature performance of the binder with biochar incorporation.*…”

8. Replace “bituminous binder” with “bitumen” or “asphalt binder” in the whole manuscript.

*Throughout the article, the term “asphalt binder” is used instead of the term “bituminous binder”.*

9. Use SI unit of Pa.s or mPa.s instead of cP in Table 1.

*Unit of Pa.s was used instead of cP in Table 1. The same corrections were made in Table 4.*

10. It is needed to precisely define fast and slow cooling condition.

"Fast and slow cooling condition" is clearly defined in the introduction section with the following statements. Additionally, schematic representations of both fast and slow cooling processes are provided in Fig 2 in Section 2.3.

*“…No detailed study has been conducted on the cooling of biochar obtained after pyrolysis. In some studies, biochar was extracted from the steel reactor only after it had cooled to room temperature following the pyrolysis process, a procedure called as slow cooling, whereas other studies did not specify the cooling method. In this study, after pyrolysis, the hot biochar was immediately removed from the steel reactor, rapidly cooled by immersing it in water at 10°C, and then dried. This process was termed rapid cooling…”*

Fig 2. (a) Rapid cooling process and (b) slow cooling process.

11. Why did the authors select three pyrolysis temperatures of 300, 450, and 600 degree of centigrade? Clarify it.

Why three pyrolysis temperatures, 300, 450 and 600 °C, were selected is explained in section 2.3. Therefore, the following sentences in red has been revised and added to section 2.3.

*“…biomass [45,46].* *In this study, the pyrolysis of industrial hemp stalks was conducted at three temperatures (300°C, 450°C, and 600°C) under two cooling methods (slow and rapid cooling) to identify the optimal production temperature and cooling conditions. To maximize biochar yield, the slow pyrolysis process was employed, with temperatures chosen within the typical range of 300–700°C for this method [6–8,47]. The pyrolysis system employed for biochar production illustrated in Fig 1. Allowing the...”*

The following references have been added to manuscript.

*6. Tan H, Lee CT, Ong PY, Wong KY, Bong CPC, Li C, et al. A Review On The Comparison Between Slow Pyrolysis And Fast Pyrolysis On The Quality Of Lignocellulosic And Lignin-Based Biochar. IOP Conf Ser Mater Sci Eng. 2021;1051: 012075. doi:10.1088/1757-899X/1051/1/012075*

*7. Ng HS, Kee PE, Yim HS, Chen PT, Wei YH, Chi-Wei Lan J. Recent advances on the sustainable approaches for conversion and reutilization of food wastes to valuable bioproducts. Bioresour Technol. 2020;302: 122889. doi:10.1016/j.biortech.2020.122889*

*47. Yadav K, Jagadevan S. Influence of Process Parameters on Synthesis of Biochar by Pyrolysis of Biomass: An Alternative Source of Energy. Recent Advances in Pyrolysis. 2020. doi:10.5772/intechopen.88204*

12. How did the authors select the biochar dosage for adding to bitumen? Clarify it.

How to select the content of biochar to be added to asphalt binder has been added to the beginning of section 2.4 as follows:

*Based on previous studies reporting that biochar content in asphalt binder ranged from 2% to 20% by weight [8], biochar concentrations of 0, 5, 10 and 15% were used in this study.*

13. It is required to specify the test temperature in which MSCR and BBR tests were performed.

In the “4.6. Multiple Stress Creep Recovery (MSCR) Test Results” section, no correction has been made since the test temperature at which MSCR was performed is stated with the following sentence.

*“MSCR test was performed on RTFO aged neat and biochar modified asphalt binders* ***at 64 °C*** *temperature at 0.1 and 3.2 kPa stress levels…”*

In the first version of the article, in the “Bending Beam Rheometer (BBR) Experimental Results” section, the experimental temperatures at which the BBR test was applied were given as follows.

“In order to evaluate the low temperature performance of bituminous binders, BBR tests were applied to PAV aged bituminous binders. Table 10 shows the creep stiffness and m-value values of bituminous binders at -6 and -12 °C temperatures and low temperature performance grade.”

*However the following change marked with red color has been made in the manuscript to correct the misunderstanding.*

*“In order to evaluate the low temperature performance of asphalt binders, BBR tests were applied to PAV aged asphalt binders at -6 and -12 °C temperatures. Table 10 shows the creep stiffness and m-value values of tested asphalt binders and, low temperature performance grade.”*

14. In Figure 8, it is needed to specify the traffic level (S, H, V, or E) of each sample. It will help the readers to better understand the difference between samples.

*The following sentence in red has been added to section 4.6 and Figure 10(b) rearranged.*

“…According to MSCR classification of neat asphalt binder, it is classified at H (Heavy Traffic Grade) level, while 300R15 asphalt binder is classified at V (Very Heavy Traffic Grade) level [40]. The corresponding traffic class for each asphalt binder type is illustrated in Fig 10b...”

Fig 10. (a) Jnr and R values at 0.1 kPa and (b) Jnr and R values at 3.2 kPa, and traffic class

15. Why did the authors consider two low-temperatures of -6 and -12 C? It is needed to measure flexural creep stiffness and m-value at lower temperatures like -18 and -24 C to better realize the effect of adding biochar to bitumen on its properties.

BBR test was performed according to the EN 14771 to determine the low-temperature stiffness of asphalt binders. According to specification (EN 14771), the maximum value of creep stiffness should not exceed 300MPa, while the minimum value of m-value was not less than 0.30 in order to avoid cracks in the pavement at very low service temperatures. As seen in Table 10, the m value at -12 ^0^C was obtained as less than 0.30 for all binder types except NB type binder. The m value at -12 ^0^C of the NB type binder was also measured as 0.30, which is the limit value. Since even smaller m values will be obtained as the temperature decreases, BBR tests were not performed at -18 and -24 ^0^C. Since the BBR measurement results at -6 and -12 ^0^C were sufficient to determine the low temperature performance grade of binder types, only the BBR test was performed at -6 and -12 ^0^C.

16. without a LCA, how did the authors claim “The findings indicate that biochar derived from industrial hemp stalks functions as a renewable, sustainable, and eco-friendly additive for bituminous binders” in the conclusion.

This study mainly investigated the determination of the optimal production temperature and cooling conditions for pyrolysis in biochar production and the utilization of biochar materials industrial hemp stalk for the modification of asphalt binder. For further studies, doing life cycle assessment for proving the sustainability aspect of industrial hemp biochar would be valuable. Therefore, the sentence “The findings indicate that biochar derived from industrial hemp stalks functions as a renewable, sustainable, and eco-friendly additive for bituminous binders” has been removed from the text.

17. The whole manuscript needs a grammatical check.

*English grammars of manuscript have been checked and modified.*

18. The reference must be cited immediately after the authors’ names. For example, in line 56, it should be Dong et al. [11].

*In the whole manuscript, the cited reference was shown immediately following the authors' names.*

*With this, we hope that the article can be published in your Journal.*

*Kind regards.*
