# Supplementary material for: Utilization of biochar derived from industrial hemp stalks with various cooling methods for asphalt binder modification
Source: PLoS One. 2025 Jul 2;20(7):e0325943. doi: 10.1371/journal.pone.0325943 (PMC12220996; doi:10.1371/journal.pone.0325943)
Supplement: Attachment — Submitted filename: Response_to_Reviewers_auresp_2.docx [file pone.0325943.s004.docx]

**Explanation of the corrections that have been made by following the reviewers comments.**

Many thanks for useful comments to our paper, and good advice for changes!

1. If the authors have adequately addressed your comments raised in a previous round of review and you feel that this manuscript is now acceptable for publication, you may indicate that here to bypass the “Comments to the Author” section, enter your conflict of interest statement in the “Confidential to Editor” section, and submit your "Accept" recommendation.

Reviewer #1: All comments have been addressed

*We would like to thank the reviewer for his/her contributions and positive responses.*

Reviewer #3: (No Response)

*We would like to thank the reviewer for his/her contributions.*

2. Is the manuscript technically sound, and do the data support the conclusions?

The manuscript must describe a technically sound piece of scientific research with data that supports the conclusions. Experiments must have been conducted rigorously, with appropriate controls, replication, and sample sizes. The conclusions must be drawn appropriately based on the data presented.

Reviewer #1: Yes

*We would like to thank the reviewer for his/her contributions and positive responses.*

Reviewer #3: Yes

*We would like to thank the reviewer for his/her contributions and positive responses.*

3. Has the statistical analysis been performed appropriately and rigorously?

Reviewer #1: Yes

*We would like to thank the reviewer for his/her contributions and positive responses.*

Reviewer #3: N/A

*We would like to thank the reviewer for his/her contributions. In the manuscript, statistical analyses were conducted using the Statistical Package for the Social Sciences to evaluate the influence of various factors on the rutting parameter (G/sinδ) at a 0.05 significance level (95% confidence level). This analysis is presented in Section 4.4.1. We have observed that Reviewer 3 selected “Yes” for the same question in his/her previous review, as shown below:*

***“2. Has the statistical analysis been performed appropriately and rigorously?'***

***Reviewer #1: Yes***
***Reviewer #2: Yes***
***Reviewer #3: Yes”***

*For this reason, we believe that the “N/A” response in the current review may have been selected inadvertently.*

4. Have the authors made all data underlying the findings in their manuscript fully available?

The [PLOS Data policy](http://www.plosone.org/static/policies.action#sharing) requires authors to make all data underlying the findings described in their manuscript fully available without restriction, with rare exception (please refer to the Data Availability Statement in the manuscript PDF file). The data should be provided as part of the manuscript or its supporting information, or deposited to a public repository. For example, in addition to summary statistics, the data points behind means, medians and variance measures should be available. If there are restrictions on publicly sharing data—e.g. participant privacy or use of data from a third party—those must be specified.

Reviewer #1: Yes

*We would like to thank the reviewer for his/her contributions and positive responses.*

Reviewer #3: No

*We would like to thank the reviewer for his/her valuable comments. All data obtained from our study are presented in the tables and figures included in the manuscript. While the data in the tables are directly accessible, to ensure public accessibility and transparency, a minimal data set containing the underlying values used to generate the figures has been submitted as a supporting file titled “Minimal Data Set.”*

5. Is the manuscript presented in an intelligible fashion and written in standard English?

PLOS ONE does not copyedit accepted manuscripts, so the language in submitted articles must be clear, correct, and unambiguous. Any typographical or grammatical errors should be corrected at revision, so please note any specific errors here.

Reviewer #1: Yes

*We would like to thank the reviewer for his/her contributions and positive responses.*

Reviewer #3: Yes

*We would like to thank the reviewer for his/her contributions and positive responses.*

6. Review Comments to the Author

Please use the space provided to explain your answers to the questions above. You may also include additional comments for the author, including concerns about dual publication, research ethics, or publication ethics. (Please upload your review as an attachment if it exceeds 20,000 characters)

Reviewer #1: (No Response)

*We would like to thank the reviewer for his/her contributions.*

Reviewer #3: Comment#3: The authors did not address the reviewer’s comment. The authors must state a specific value or range for “negative effect on low-temperature performance of the binder.” Instead of presenting some samples of articles, the authors must focus on addressing the reviewer’s comment properly.

*We would like to thank the reviewer for his/her contributions. In response to the reviewer's comment, the last part of the Abstract has been revised as follows:*

“…temperature of 300°C and rapid cooling. The low-temperature performance grade of biochar-modified asphalt binders decreased by one grade, while the high-temperature performance grade increased by at least one grade. The addition of biochar was shown to have a negative impact on the binder’s low-temperature performance. Based on these findings, it was concluded that the biochar additive, derived from industrial hemp stalk, is particularly well-suited for regions characterized by hot climates.”

*With this, we hope that the article can be published in your Journal.*

*Kind regards.*
